# Predictors of life satisfaction among Asian American adolescents- analysis of add health data

**DOI:** 10.1186/s40064-015-1008-5

**Published:** 2015-05-06

**Authors:** Jui-Yen Huang, Kuan-Yuan Wang, Tamar Ringel-Kulka

**Affiliations:** Department of Pediatrics, Kaohsiung Medical University Hospital, Kaohsiung Medical University, No.100, Tzyou 1st Road, Kaohsiung, 807 Taiwan; Department of Maternal and Child Health, Gillings School of Global Public Health, University of North Carolina- Chapel Hill, CB# 7445, 404A Rosenau Hall, 421 Pittsboro Street, Chapel Hill, NC 27599-7445 USA; Division of Geriatrics and Gerontology, Department of Internal Medicine, Kaohsiung Medical University Hospital, Kaohsiung Medical University, No.100, Tzyou 1st Road, Kaohsiung, 807 Taiwan

**Keywords:** Asian American, Adolescent, Global life satisfaction, Self-esteem, Peer support

## Abstract

Life satisfaction correlates with adolescent risk taking behavior and their outcomes in adulthood. Despite the fast rise in numbers of Asian adolescents in the U.S., the predictors of their life satisfaction are not well understood. This study examined the relationship between several demographic and contextual factors and global life satisfaction among this population. Data were derived from the National Longitudinal Study of Adolescent Health (Add Health), a nationally representative probability sample of US adolescents. Bivariate and multivariable logistic regression was conducted to evaluate hypothesized predictors of global life satisfaction of Asian American adolescents. All analyses were conducted using STATA version 11. After exclusion of cases with missing values, 1021 Asian American adolescents were studied. Self- rated health, self-esteem, perceived neighborhood quality, parental support and peer support were significantly and positively related to better global life satisfaction. However, after controlling for other factors, only self-esteem (adjusted odds ratio [aOR]: 4.76; 95% confidence interval [CI]: 2.86-8.33) and perceived peer support (aOR: 2.76; 95% CI: 1.33-5.76) significantly predicted higher life satisfaction. Peer support and adolescents’ self-concept are strongly correlated with Asian American adolescents’ subjective well-being. To promote the wellness of this population, culturally sensitive strategies in developing peer relationship and healthy self-concept may be effective. More studies are needed for subgroup comparison of various ethnicities among Asian American adolescents.

## Introduction

Adolescents (ages 10 to 19) make up 13.4 percent of the population of the United States (Age and Sex Composition in the United States 2012). Their current health status and future quality of life are greatly influenced by the behavioral patterns formed at this developmental period (Lawrence et al. 2009).

Life satisfaction, a cognitive aspect of subjective well-being (Diener 1994), has been found to correlate with adolescent risk-taking behaviors. A higher level of life satisfaction is associated with lower violence, less suicide attempts, fewer sexual risk-taking behaviors and diminished substance abuse among adolescents (Sun and Shek 2010; Valois et al. 2002; MacDonald et al. 2005). When undergoing significant life events, life satisfaction plays as a protective psychological strength in the process of adaptive development and prevents adolescents from developing externalizing psychopathological behaviors (Suldo and Huebner 2004). Such beneficial effects may extend to their adult life. Studies have shown that happiness leads to positive health, behavioral, psychological, and social outcomes in adulthood (Lyubomirsky et al. 2005). Therefore, enhancing life satisfaction during adolescence is a cornerstone of promoting healthy development.

Although the life satisfaction of adolescents has drawn more attention and many studies have been done during the past decade (Proctor et al. 2009), most of the studies for the US population were addressing White American and African American adolescents. Studies have revealed variations in the contribution of specific contextual and individual factors to the perceived life satisfaction among different sociocultural groups (Bradley and Corwyn 2004; Schwarz et al. 2012). Such variations have also been observed in European adolescents (Cacioppo et al. 2013; Mahmud and Schölmerich 2011). Various levels of acculturation among immigrants were associated with less life satisfaction, compared with native residents. As the US adolescent population becomes more racially and ethnically diverse, the number of Asian American adolescents is rapidly growing. In 2012, more than 17 percent of U.S adolescents age 10 to 19 are Asian alone or in combination (Race - The Asian Alone or in Combination Population in the United States 2012). Consequently, it is important to identify the predictors of life satisfaction within this unique population so as to inform culturally sensitive interventions. The existing literatures about Asian adolescents’ life satisfaction mostly investigated those residing in Asian countries (Proctor et al. 2009). The outcomes of such studies cannot be applied to Asian American adolescents because of the complex differences in socio-cultural contexts. Moreover, most existing studies of life satisfaction that have included Asian American youth have small and often convenience samples of uncertain generalizability (Bradley and Corwyn 2004).

**Figure 1:**
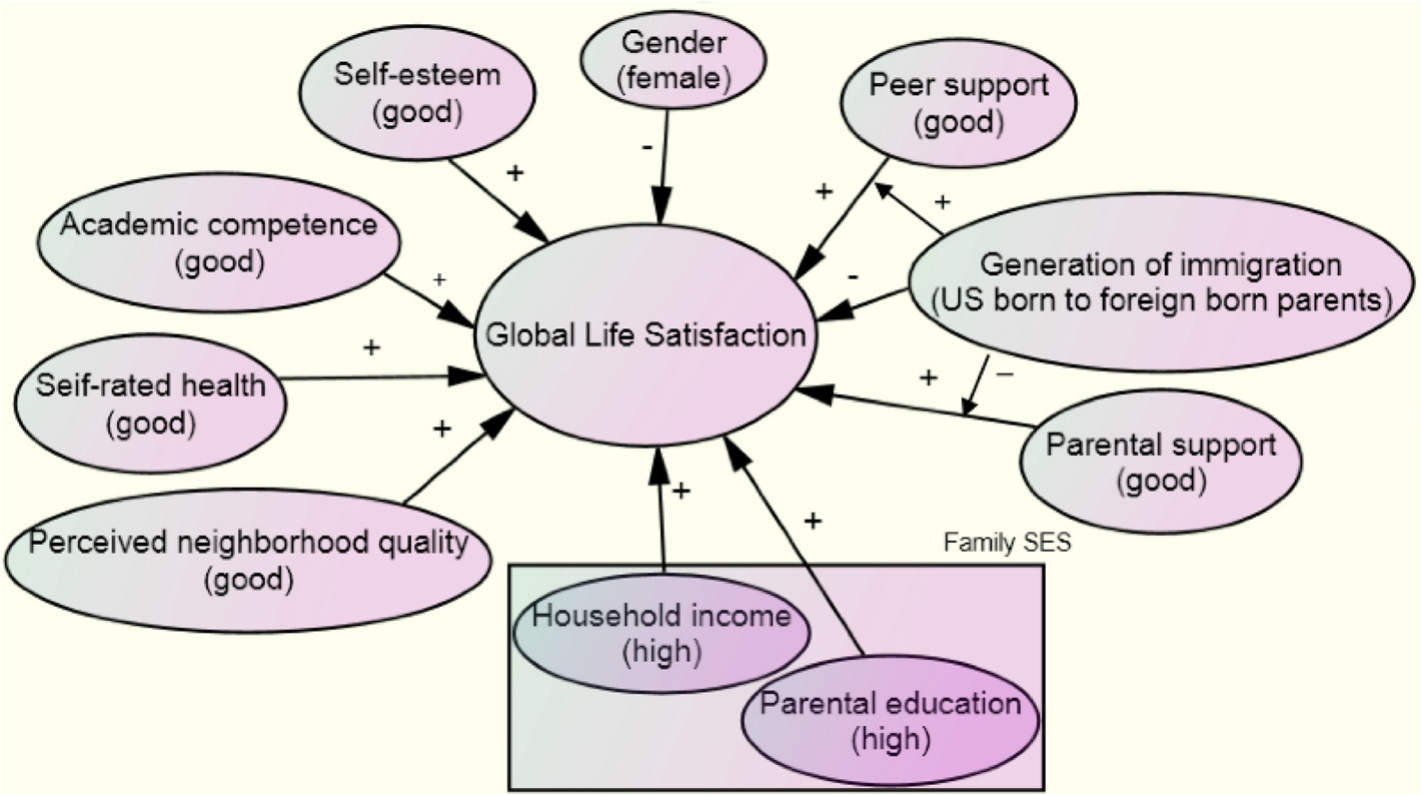
Conceptual model. A “+” sign indicates a hypothesized positive association. A “-“ sign indicates a hypothesized negative association.

The purpose of this study is to understand the roles of several individual factors (gender, self-rated health, self-esteem, academic competence and generation of immigration), environmental factors (socioeconomic status, and perceived neighborhood quality) and social factors (parental support and peer support) in predicting the global life satisfaction of Asian American adolescents by analyzing a nationally representative probability sample. The conceptual model (Figure 1) guiding this study is rooted in existing research on adolescents’ life satisfaction, particularly among Asian Americans. In this model, Asian American girls are hypothesized to have lower life satisfaction than boys due to more contrary expectations they may experience among peers and from parents (Hondagneu-Sotelo 2003; Chris et al. 2006). Those who have better self-rated health status, self-esteem or academic competence are hypothesized to have better life satisfaction (Proctor et al. 2009; Zullig et al. 2005; Diener and Diener 1995; Leung et al. 2004). First generation US- born adolescents (whose parents are foreign- born) and first generational immigrant adolescents who had resided in the US for more than 10 years are hypothesized to experience greater acculturation gap, intergenerational conflicts and lower life satisfaction than their counterparts (Juang and Nguyen 2011; Birman 2006). Furthermore, those who are from families with higher socioeconomic status are hypothesized to have better life satisfaction (Acock and Kiecolt 1989). Adolescents who perceive better neighborhood quality, including safety and cohesion, are hypothesized to be more satisfied with their life (Curtis et al. 2004). Lastly, those who perceive better social support, which includes parental support and peer support, are hypothesized to have better life satisfaction (Schwarz et al. 2012; Piko and Hamvai 2010). Generation of immigration may moderate the associations between peer/parental support and level of life satisfaction.

Despite the rise in their numbers, there is limited data and understanding of life satisfaction in Asian American adolescents. Our study aims to explore the relationship between extensive demographic and contextual factors and global life satisfaction among this specific population using nationally representative probability sample.

## Materials and methods

### Data

The current research is based on Wave I contractual data from the National Longitudinal Study of Adolescent Health (Add Health 2014), a cohort study using a nationally representative probability sample of 7th through 12th-grade U.S. adolescents and the multiple social contexts of their life since 1994–95. The detailed description of Add Health study is available at http://www.cpc.unc.edu/projects/addhealth. The study in Wave 1 mainly included 4 parts: School Administrator Questionnaire, Adolescent In-School Questionnaire, Parent In-Home Questionnaire and Adolescent In-Home Interview.

The present study focuses on Asian and Pacific Islander American respondents who participated in the in-school questionnaire and in home survey. Asian and Pacific Islander Americans were identified mostly by self-report. If the adolescent self-report was missing, interviewer report was consulted. 1585 Asian and Pacific Islander Americans were identified by the criteria. However, the final sample was reduced to 1021participants because of missing values. The present study was determined to be exempt from oversight by the Public Health-Nursing Institutional Review Board at UNC-Chapel Hill.

### Measures

#### Dependent variable

In this study, the dependent variable, global life satisfaction, was assessed by a single item asking the adolescents “How often was the following true during the last week? You were happy”. Responses to the question were used to create a dichotomous variable (never, rarely or sometimes felt happy, recoded as “0” = low life satisfaction; felt happy a lot of the time, most of the time or all the time, recoded as “1” = high life satisfaction). Those who refused or answered “don’t know” were coded as “missing”.

#### Independent variables

A detailed description of measures for independent variables follows.

#### Gender

The data of gender were self-reported in in-school questionnaire. Male was coded 0 and female was coded 1. People who refused to answer and who answered “don’t know” were coded as “missing”. For those with missing data, interviewer report was consulted.

#### Generation of immigration

Generation of immigration was categorized based on the birthplace of respondents and their parents and the duration of residency in the U.S., if the respondents were not born in the U.S., in order to reflect the potential acculturative gap between the youth and their parents. The information was collected mainly from student in-home survey. For those with missing value for parental birthplace, parent interview was consulted. Respondents who moved to the U.S. before their first birthday were recoded as native-born. Four categories were created using this strategy: first generation (foreign born to at least one foreign born parent) with less than 10 years residency in the U.S., coded as “1”; first generation (foreign born to at least one foreign born parent) with more than 10 years residency in the U.S., coded as “2”; second generation (U.S. born to at least one foreign born parent), coded as “3”; and third + generation (U.S. born to U.S. born parents) youth, coded as “4”.

#### Academic competence

We measured academic competence by averaging students’ self-reported grades for 4 subjects (English, Math, History, and Science) from adolescent in-home interview. For those with missing value in in-home interview, in-school questionnaire was consulted. Numbers were used for representing the letter grade. For example, “1” represented “A” and “4” represented “D”. Mean score of our sample was calculated and used as the criteria to categorize good and poor academic performance. Those with averaged score above the mean were coded as “1”: good academic competence. For those with averaged score below the mean were coded as “2”: poor academic competence.

#### Self-esteem

Adolescent self-esteem was assessed by one question “You like yourself just the way you are”. Those who expressed “strongly agree” or “agree” were coded as “1” = having good self-esteem. On the other hand, those who answered “neither agree nor disagree”, “disagree” or “strongly disagree” were coded as “2” = having poor self-esteem.

#### Self-rated health

Self-rated health was measured by using one question “In general, how is your health?” The responses were sorted into two categories: “excellent, very good, or good”, recoded as”0” = good self-rated health; “fair or poor”, recoded as “1” = poor self-rated health.

#### Family socioeconomic status

Measures of SES were drawn from information obtained during the parental interview. Household income and parental educational attainment were both used as indicators of family SES. The mean value of overall 1994 household income before taxes in the sample was calculated and used as a standard for categorization. Two categories were created, including “0” = household income less than mean value; “1” = household income higher than mean value. Parental educational attainment was also reported in the parental interview. The higher level attained by either the resident mother or father was used to create a 4-level ordinal variable as described in previous analyses that used Add Health data (Hussey et al. 2007). Categories included less than high school, coded as “0”; high school degree, general equivalency degree (GED), or vocational training instead of high school, coded as “1”; vocational training after high school or some college, coded as “2”; college graduate and higher, coded as “3”.

#### Perceived neighborhood quality

Neighborhood quality was measured by 2 questions asking the students’ perceived neighborhood safety and cohesion: “People in this neighborhood look out for each other” “Do you usually feel safe in your neighborhood?” Those answered “no” for both questions were categorized as “having poor perceived neighborhood quality” and coded as “0”. On the other hand, those who answered “yes” to at least one of the questions were categorized as “having good perceived neighborhood quality” and coded as “1”.

#### Parental support

Parental support was measured by using one question “How much do you feel that your parents care about you?” Two categories were created according to the responses: “not at all, very little or somewhat”, recoded as “0” = poor perceived parental support; ” quite a bit or very much”, recoded as “1” = good perceived parental support.

#### Peer support

Level of perceived peer support was assessed by an item asking “How much do you feel that your friends care about you?” A dichotomous variable was created as follows: “not at all, very little or somewhat”, recoded as “0” = poor perceived peer support; ” quite a bit or very much”, recoded as “1” = good perceived peer support.

### Analytic techniques

All Analyses were conducted using STATA version 11 Statistical Software. In order to adjust for the complex clustered sampling frame of Add Health and ensure that the results are nationally representative, survey commands and sampling weights were used. Since the dependent variable was dichotomous, binary logistic regression was conducted to analyze the relationships between predicting factors and level of life satisfaction. Multivariable logistic regression, which adjusted for gender, self-rated health, generation of immigration, self-esteem, academic performance, peer support, parental support, perceived neighborhood quality, household income and parental educational attainment, was also performed. Significance levels were set at p < 0.05.

Potential interactions between generation of immigration and each of the 2 social factors (parental support and peer support) were examined separately in unadjusted models predicting level of life satisfaction. These interaction terms were both insignificant and were not included in the multivariate model.

## Results

Table 1 reports the demographic characteristics of our sample of Asian American adolescents. In the weighted sample of Asian American adolescents, 51.5% were male. The mean age of the population was 16.06 years. The ethnicity background for these Asian American adolescent subjects included Chinese (12.8%), Filipino (32.9%), Korean (9.8%), Japanese (7.6%), Asian Indian (3.4%), Vietnamese (9.8%) and others (23.5%). Slightly less than half (44.6%) of the population were born in the U.S. The majority (68.5%) of the subjects came from families whose annual incomes were higher than 45,700 USD, the average household income. More than half (51.4%) of the adolescents had highly educated parents (college graduate or higher). 75.2% of the subjects reported high life satisfaction.

**Table 1:** Survey-weighted characteristics of sample participants of Asian American adolescents (N == 1,585)*

**Characteristic**	**Percentage** ^**†**^
**Age**	
Mean (SE)§	16.06 (0.08)
**Gender**	
Male	51.5
Female	48.5
**Asian background**	
Chinese	12.8
Filipino	32.9
Japanese	7.6
Asian Indian	3.4
Korean	9.8
Vietnamese	9.8
Other	23.5
**Immigration status**	
Foreign born to foreign born parent & residency ≤ 9 years	25.1
Foreign born to foreign born parent & residency ≥ 10 years	20.2
U.S. born to at least one foreign born parent	27.2
U.S. born to U.S. born parents	17.4
Missing	10.1
**Household income**	
Lower than average (45,700 USD/year)	31.5
Higher than average	68.5
**Parental education**	
Less than high school	9.7
High school/GED/Vocational	18.6
Some college	17.8
College graduate and higher	51.4
Missing	2.5
**Life satisfaction**	
Low	24.7
High	75.2
Missing	0.1

*Number of subjects is unweighted.

^†^Percentages and mean are weighted.

^§^SE: Standard error.

### Individual factors

**Figure 2:**
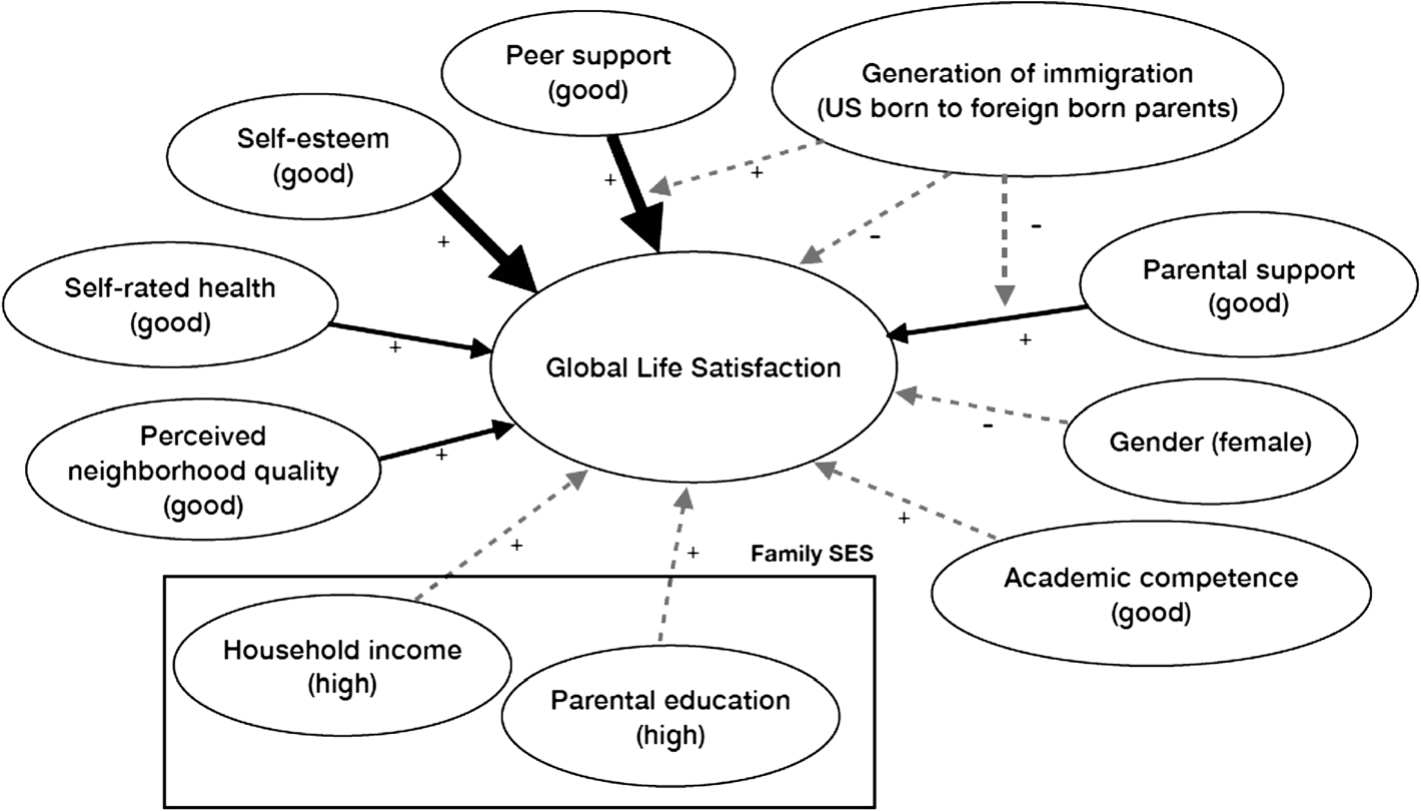
Test results of the conceptual model. Bold arrows indicate significant associations in multivariate model. Thin arrows indicate significant associations in bivariate model, but non-significant in multivariate model. Dotted arrows indicate non-significant associations in both models.

When investigating gender, self-rated health, self-esteem, academic competence and generation of immigration as predictors of life satisfaction in our targeted populations as hypothesized (Figure 2), we found that self-esteem was a significant predictor in both bivariate and multivariate model (Table 2). Asian American youth who had good self-esteem (77.9%) were 4 times as likely to experience high life satisfaction than those with poor self-esteem (OR poor/good =0.25; 95% CI: 0.16-0.38). This relationship was still notable after adjusting for other factors (aOR = 0.21; 95% CI: 0.12-0.35). Moreover, those who reported poor health (6.3%) were less likely to have high life satisfaction (OR = 0.3; 95% CI: 0.16-0.57) than their counterparts in the bivariate analyses. This relationship was not remarkable in the multivariate model though.

**Table 2:** **Odds ratios (and 95% confidence intervals) from logistic regression models assessing associations between selected characteristics and adolescent perceived life satisfaction**

**Characteristics**	**Bivariate analyses**	**Multiple logistic regression**
**Gender**		
Male (ref)	1.00	1.00
Female	0.99 (0.66-1.47)	1.44 (0.83-2.52)
**Self-rated health**		
Good (ref)	1.00	1.00
Poor	0.30 (0.16-0.57)***	0.77 (0.31-1.91)
**Generation of immigration**		
Foreign born to foreign born parent & residency ≤ 9 years (ref)	1.00	1.00
Foreign born to foreign born parent & residency ≥ 10 years	0.83 (0.45-1.54)	0.54 (0.24-1.19)
U.S. born to at least one foreign born parent	1.15 (0.66-1.99)	1.05 (0.54-2.7)
U.S. born to U.S. born parents	1.21 (0.65-2.26)	1.15 (0.53-2.46)
**Academic competence**		
Good (ref)	1.00	1.00
Poor	0.66 (0.42-1.05)	0.65 (0.37-1.13)
**Self-esteem**		
Good (ref)	1.00	1.00
Poor	0.25 (0.16-0.38)****	0.21 (0.12-0.35)***
**Household income**		
Low (ref)	1.00	1.00
High	0.78 (0.50-1.21)	0.96 (0.54-1.70)
**Parental education**		
Less than high school (ref)	1.00	1.00
High school/GED/Vocational	1.13 (0.53-2.45)	1.76 (0.66-4.68)
Some college	1.97 (0.85-4.56)	1.93 (0.70-5.35)
College graduate and higher	1.81 (0.91-3.62)	2.43 (1.04-5.69)*
**Perceived neighborhood quality**		
Poor (ref)	1.00	1.00
Good	2.01 (1.34-3.01)***	1.60 (0.92-2.79)
**Parental support**		
Poor (ref)	1.00	1.00
Good	2.67 (1.26-5.65)**	1.26 (0.44-3.62)
**Peer support**		
Poor (ref)	1.00	1.00
Good	2.71 (1.66-4.42)****	2.76 (1.33-5.76)**

*p < .05; **p < .01; ***p < .001; ****p < .0001. Notes: ref = reference group.

Furthermore, there was no significant difference in life satisfaction between male and female Asian American adolescents. The difference in life satisfaction among various generations of Asian American youths was not remarkable, either. Academic competence, although highly valued in Asian culture, was not a significant predictor in Asian American adolescents’ life satisfaction (OR = 0.66; 95% CI: 0.42-1.05).

### Environmental factors

Family socioeconomic status, which was measured by household income, and parental educational attainment and perceived neighborhood quality were the environmental predictors studied. No significant relationship was found between household income and adolescent life satisfaction (p = 0.267). Higher parental educational level was not significantly related to higher adolescent life satisfaction in the bivariate analyses, either. However, after controlling for other predictors, adolescents whose parents were highly educated (college graduate or higher) were more likely to have higher life satisfaction than others (aOR = 2.43; 95% CI: 1.04-5.69). Neighborhood quality is also an important factor for adolescent life satisfaction. In the bivariate analyses, adolescents who perceived good neighborhood quality, such as neighborhood cohesion and safety, were twice as likely to experience high life satisfaction than those who perceived poor neighborhood quality (OR = 2.01; 95% CI: 1.34-3.01). Nevertheless, the association was not significant after controlling for other factors (aOR = 1.6; 95% CI: 0.92-2.79).

### Social factors

In this study, two potential social predicting factors of adolescent life satisfaction were investigated: parental support and peer support. As expected, peer support was found a significant predictor for life satisfaction among Asian American adolescents in both bivariate (p < 0.0001) and multivariate (p < 0.01) analyses. Adolescents who perceived good peer support (81.5%) were 2.7 times likely to have high life satisfaction, compared to their counterpart (aOR = 2.76; 95% CI: 1.33-5.76). According to the survey, 93.3% of the Asian American adolescents perceived good support from their parents. In the bivariate analyses, they were 2.67 times likely to experience high life satisfaction than those with poor parental support (OR = 2.67; 95% CI: 1.26-5.65). However, this positive association was found statistically insignificant after adjusting for other predictors (aOR = 1.26; 95% CI: 0.44-3.62).

## Discussion

Using a nationally representative probability sample of Asian American adolescents, the purpose of this study was to examine the plausible predicting factors of global life satisfaction for this specific population. Based on the results, it can be concluded that perceived peer support and self-esteem play a remarkable role in Asian American adolescents’ life satisfaction. The contribution of gender, generation of immigration, academic competence, and household income to life satisfaction in Asian American adolescents, on the other hand, is negligible. Other factors such as self-rated health, perceived neighborhood quality and parental support are important factors however they are not a significant predictor by themselves. The findings is inconsistent with previous literature by Bradley and Corwyn (Bradley and Corwyn 2004), who found parental marital status and home physical environment significant predictors of life satisfaction for Chinese American adolescents, while self-esteem and income-to-needs were important, but non-significant factors. Nevertheless, peer group experience and neighborhood quality were not included in their analyses.

The main strength of this study is the usage of nationally representative probability sample from Add Health, which may generate results of better validity and generalizability. As to our knowledge, this study has the largest sample population in studying the predictors of life satisfaction among Asian American adolescents. Another strength of this study is the extensive exploration of potential predictors for adolescent life satisfaction while weighing the difference between Asian and American culture.

However, there are several limitations for this study. First, the cross-sectional measure of predicting factors and perceived life satisfaction allows for no conclusions about the direction of the association. Also, the measures of variables, including the predicting factors and life satisfaction, are self-reported, single- item questions, instead of multiple items scales. These measures may cover a variety of transient reactions. According to previous publications, such single item, domain-free measure of life satisfaction is judged by individuals’ personal standards and is termed “global life satisfaction” (Antaramian et al. 2008). When compared with multidimensional scales, a correlation of 0.61-0.62 has been reported among elementary school students and grades 3–8 Canadian children (Huebner 2004). Another limitation concerns the within- group diversity, which was not analyzed in this study. Asian population in the U.S. are very heterogeneous socially, culturally and economically (Rhee 2009). Therefore, Rhee (Rhee 2009) commented that “it is difficult to generalize the life experiences of diverse Asian American groups, because they all have different social, cultural, and linguistic backgrounds, and also because their experiences and expectations change differentially as the length of residency in the United States increases” (p. 83).

## Conclusions

With relatively higher educational, and economic accomplishments and lower criminal rate, Asian Americans have been pictured as the “model minority” for several decades in the U.S. society (Lee et al. 2009). However, some mental health problems and social adjustment issues among this population have drawn attention on the media recently (Rhee 2009). This study provides a convenient tool for primary care providers or school counselors to early identify those who are at high risk of poor subjective well-being among Asian American adolescents and formulate appropriate services. Interventional programs that promote positive youth development would be most effective if they are culturally responsive. According to the results, peer support and self-esteem play the most crucial roles in Asian American adolescents’ life satisfaction. It is thus likely that the promotion of well-being among this population requires additional efforts to improve their social environment and self-concept so as to promote positive youth development (Guerra and Bradshaw 2008). Strategies such as promoting personal skills in building peer relationships, formulating peer group at schools or classes for promoting positive sense of self may be helpful in this case.
